# Ethical considerations related to participation and partnership: an investigation of stakeholders’ perceptions of an action-research project on user fee removal for the poorest in Burkina Faso

**DOI:** 10.1186/1472-6939-15-13

**Published:** 2014-02-20

**Authors:** Matthew R Hunt, Patrick Gogognon, Valéry Ridde

**Affiliations:** 1 School of Physical and Occupational Therapy, McGill University, Montreal, Canada; 2 Centre for Interdisciplinary Research in Rehabilitation, Montreal, Canada; 3 Department of Social and Preventive Medicine, University of Montreal School of Public Health, Montreal, Canada; 4 Research Centre of the University of Montreal Hospital Centre (CRCHUM), Montreal, Canada

**Keywords:** Action-research, Burkina Faso, Ethics, Participatory research, Partnership, Research ethics, User fees

## Abstract

**Background:**

Healthcare user fees present an important barrier for accessing services for the poorest (*indigents*) in Burkina Faso and selective removal of fees has been incorporated in national healthcare planning. However, establishing fair, effective and sustainable mechanisms for the removal of user fees presents important challenges. A participatory action-research project was conducted in Ouargaye, Burkina Faso, to test mechanisms for identifying those who are indigents, and funding and implementing user fee removal. In this paper, we explore stakeholder perceptions of ethical considerations relating to participation and partnership arising in the action-research.

**Methods:**

We conducted 39 in-depth interviews to examine ethical issues associated with the action-research. Respondents included 14 individuals identified as indigent through the community selection process, seven members of village selection committees, six local healthcare professionals, five members of the management committees of local health clinics, five members of the research team, and four regional or national policy-makers. Using constant comparative techniques, we carried out an inductive thematic analysis of the collected data.

**Results:**

The Ouargaye project involved a participatory model, included both implementation and research components, and focused on a vulnerable group within small, rural communities. Stakeholder perceptions and experiences relating to the participatory approach and reliance on multiple partnerships in the project were associated with a range of ethical considerations related to 1) seeking common ground through communication and collaboration, 2) community participation and risk of stigmatization, 3) impacts of local funding of the user fee removal, 4) efforts to promote fairness in the selection of the indigents, and 5) power relations and the development of partnerships.

**Conclusions:**

This investigation of the Ouargaye project serves to illuminate the distinctive ethical terrain of a participatory public health action-research project. In carrying out such projects, careful attention and effort is needed to establish and maintain respectful relationships amongst those involved, acknowledge and address differences of power and position, and evaluate burdens and risks for individuals and groups.

## Background

In the 1980s, user fees for access to health care services were introduced in many countries with the support of the World Bank, including almost all countries in sub-Saharan Africa [1]. These payment systems, however, contributed to difficulties for the most vulnerable in these countries to access healthcare, leading to decreased use of services among these groups [2]. In 1987 the Bamako Initiative was established in order to address both the quality, as well as the accessibility of healthcare [3]. The Bamako Initiative proposed specific approaches for establishing community financing for health services, and made recommendations to ensure that those who were very poor were not excluded by this system. However, implementation of the Bamako recommendations was partial, and these equity promoting mechanisms were not established in many settings, resulting in continued inequality in access to healthcare [4,5]. One approach to address this concern has been the removal of user fees for select groups such as the poorest individuals in a community [6]. Where user fee abolition is intended to target the most vulnerable, a particular challenge is raised: how to define and identify who should qualify in a context of widespread poverty [7]. This is an important question for policy-makers seeking to address a major health equity gap [8].

Burkina Faso is a very poor country and was ranked 183 out of 186 countries on the 2012 Human Development Index [9]. In 2009, 44.6% of the population lived on less that 1.25$/day [10]. As one of the countries in sub-Saharan Africa where healthcare user fees are in place and efforts are being made to address equity in access to healthcare services, policy makers have sought to alleviate this burden by eliminating user fees for the poorest [11]. The national health policy indicates that the poorest individuals, termed “*indigents”*, should have access to free healthcare; however, this provision of the policy has yet to be enacted [12]. One explanation for this situation is the lack of reliable mechanisms for identifying those individuals who are indigent, i.e. those who experience a long-standing inability to access healthcare due to their situation of extreme poverty [6].

In order to evaluate mechanisms to identify who should be identified as indigent and thus benefit from user fee removal, a team of researchers from Burkina Faso and Canada, in collaboration with policy-makers, local officials and community members, initiated an action-research project in Ouargaye District. This is a rural region (96% of the 260,000 population) where subsistence agriculture predominates. The project carried out in Ouargaye involved the implementation and testing of mechanisms for identifying those who are indigent, and models for subsidizing the exemption of user fees. The action-research has been described in detail elsewhere [13]. Briefly, the project employed a participatory, community-based action-research model. The action component involved the implementation of a mechanism to identify indigent individuals and provide them with free access to healthcare. Removal of user fees was covered by endogenous funding; the marginal profit generated from the sale of medications and consultation fees in local health dispensaries was used to cover the health care costs of individuals identified as indigent. This funding mechanism was consistent with provisions within the national health policy of Burkina Faso, but which had not been applied.

Rather than implementing a set of pre-determined criteria to be used when individuals present to the health clinic, the process employed in Ouargaye was a mechanism of selection carried out by a committee of village representatives. Village selection committees (VSCs) had the responsibility to generate a list of indigent individuals in their community based on a consensually-generated definition of indigence. The list was subsequently ratified by the local chief. Final review and approval of the list was made by the Management Committee (MC) of the local health clinic. The MC, made up of community members, reviewed this list with the goal of confirming whether those selected were truly the poorest in the village. Those who were eventually selected as indigents were then issued an official indigence card by the governmental authorities that would enable them to have free access to see a primary healthcare provider and be exempted from costs for prescribed medications from the local dispensary.

The research component of the project aimed to evaluate the intervention across multiple domains: effectiveness, equity, implementation, process, sustainability and ethics [14-16]. These evaluations began concurrently with the action component and continued after its completion. The evaluation related to ethics was conducted 4 years after the beginning of the action component [17]. In this article, we present and discuss stakeholder perceptions of ethical considerations relating to partnership and participation in the project.

Participatory and action-research approaches to inquiry are closely associated with social justice, as they seek to address health needs of marginalized groups through collaborative processes [18,19]. These approaches also raise a range of ethical issues. Action-research and participatory research are predicated upon partnerships between different groups of stakeholders with the goal of generating knowledge that can guide improvement of practices or policies in particular settings [20,21]. Given the diversity of actors involved in these activities, negotiating expectations and roles related to decision-making and the sharing of tangible and intangible benefits of research can be sources of uncertainty or conflict [22]. These considerations relate to the complex power relations that are involved, and which are compounded by cultural, social and institutional differences between groups [23]. Achieving effective and respectful partnership in such projects is an inherent challenge. In some circumstances, merely “token” partnerships may be instituted or significant differences of power and privilege may remain unaddressed and result in stunted collaboration [24,25].

In working with communities and other partners, questions may be raised regarding the ownership of and control over study results, including the response to unflattering or negative research findings [26,27]. Where research participants are especially vulnerable due to situations of marginalisation or deprivation, the call for participatory approaches may be particularly persuasive but the risks will also be amplified [20]. Such risks may accrue for both individuals and groups, and both individual and communal protections may be warranted, as well as careful reflection for how to adjudicate between individual and collective risks [21]. Underlying these diverse ethical questions is the importance, and challenge, of establishing respectful and effective partnerships and participation through which to address them [28].

## Methods

We conducted a qualitative study based on interpretive description methodology [29]. Interpretive description is a methodological framework developed within applied health disciplines to address the complex experiential issues arising in health care and health services. This approach is grounded in a constructivist and naturalistic orientation to inquiry, and aims to develop a coherent conceptual account of patterns and commonalities that characterize a phenomenon, while accounting for individual variations [30]. In this paper we present findings related to stakeholder perceptions of ethical considerations and efforts undertaken to address these issues. The concept of ‘ethical consideration’ was broadly construed in this inquiry, and encompassed features of the project in which values that were important to respondents were experienced as being realized or thwarted. This broader scope of inquiry draws upon a moral experience framework for empirical bioethics that extends beyond a narrow focus on ethical quandaries or problems [31].

### Respondents

39 respondents were interviewed for the ethics analysis: 14 individuals identified as indigent through the community selection process, seven members of VSCs, six local healthcare professionals, five members of MCs of local health clinics, five members of the research team, and four regional or national policy-makers. All respondents in the ethics evaluation were previously involved in the broader action-research program. Individuals were invited to participate in the interview for the ethics evaluation through invitation by the project’s research coordinator. Purposive sampling was employed to recruit a diverse group of respondents from across the various sub-groups involved in the project. Pragmatic considerations related to availability and geography also guided recruitment. An informed consent process specific to this evaluation component was undertaken.

### Data collection and analysis

Semi-structured, in-depth interviews were conducted with each respondent based on an interview guide. Interviews were audio-recorded and transcribed. Interviews were conducted in French or Yana, the local language. A local research assistant who is fluent in Yana conducted these interviews, and subsequently translated the transcripts into French for inclusion in the data analysis. This individual did not take part in other aspects of data collection or analysis during the Ouargaye action-research.

Once transcription was completed, a short synopsis of each interview was written. Three members of the research team collaboratively developed a preliminary coding scheme, and coding was organized using NVivo software [32]. Constant comparative techniques were employed to compare coded data within a single transcript and across transcripts. Attention to the role of individual respondents within the project (e.g. policy maker, health professional, etc.) was retained during this comparison process. Conceptualization of relationships between codes was recorded in theoretical memos. Through this process we sought to develop broader concepts and specify their properties, returning repeatedly to the data to test their development [33]. An inductive approach was used to develop themes that emerged from the data, rather than applying a preselected framework or conceptual model. The development of these inductively derived themes was undertaken by two researchers not involved in other aspects of the action-research or its evaluation (*MH and PG*), and reviewed with a third researcher (*VR*) who was involved throughout the action-research.

The study was approved by the Research Ethics Board of the Research Centre of the Centre Hospitalier de l’Université de Montreal and the National Research Committee of Burkina Faso.

## Results

The Ouargaye project was based on a participatory approach, included both implementation and research components, and focused on a vulnerable group within small, rural communities. These features of the project were associated with a range of ethical considerations. Five inductively derived themes were developed that illuminate key facets of how stakeholders perceived and experienced ethical considerations related to partnership and participation in the action-research: 1) seeking common ground through communication and collaboration, 2) community participation and the risk of stigmatization, 3) impacts of local funding of user fee removal, 4) efforts to promote fairness in the selection of the indigents, and 5) power relations and the development of partnerships. We include selected quotations to illustrate aspects of the analysis. Translation of these quotations from French into English was undertaken by an independent professional translator.

### Seeking common ground through communication and collaboration

The action-research in Ouargaye was designed as a community-based project and relied upon the participation and engagement of diverse actors, including local community members. The participatory model did not extend across the full life cycle of the project, however. Initial phases of the project including its conception, applications for funding, and selection of the research sites were undertaken by researchers from Burkina Faso and Canada, and subsequently involved national and regional policy-makers. Community involvement began with decision-making around how the indigents would be selected, including evaluative criteria and establishment of an operational definition of indigence: “someone who is extremely disadvantaged socially and economically, unable to look after himself (herself) and devoid of internal or external resources.” A key concern for respondents from amongst the research team and policy-makers was the possibility of establishing a shared vision for the project and for local communities to accept to take part. At the same time, there were questions raised about the need to ensure that methodological rigor was maintained and logistical aspects were addressed. Challenges around developing common understanding and shared commitment for the project were also described by several respondents from the local communities, including members of the VSCs and MCs, as well as health workers. The degree of community engagement also differed between the action (selection of the indigents) and research (evaluation) components. While the action component was more broadly shared with the community, decisions and implementation of the research components of the project remained more within the control of researchers.

Diverse community members participated in the planning activities for how the action-research would be established in their villages and input was sought from across different groups in the villages. In describing the process of engaging the local communities, a local health worker reported: “We took all the social layers. We involved the elected officials, religious leaders, community leaders, women…” In these ways, diverse representatives of the community helped to shape the practical steps that would be implemented in the project. Engaging community participation was, however, described as challenging including communication and comprehension of the project, and understanding of the financial implications of the funding model for the village pharmacies.

Planning workshops in the local communities resulted in the establishment of a selection process that would be primarily enacted by community members. It relied upon the sequenced participation of multiple groups in the community: VSCs, MCs of the health clinics, and traditional chiefs, as well as health workers who resided in the community. Given the multiple groups participating in the project, there were obstacles to clearly communicating the purpose and methods of the project.

Effective communication was described by respondents as key for establishing a shared understanding of the project and necessary for community participation. A policy-maker reported that “the main challenge was understanding of the project… Would everyone accept it?” Steps such as regular meetings with village members and the frequent presence of project staff in the villages helped improve understanding, however comprehension remained a challenge. One of the indigents reported that though she had herself benefited from free healthcare, she only understood how she was selected when she watched the film “Ah, les indigents” which had been developed as a modality for knowledge transfer about the project and used as a means of sharing study findings with the community after the completion of the project (available at: http://www.youtube.com/watch?v=MnUTi3FwIkc). Respondents, including members of the MCs and VSCs, reported that many members of the community had a limited understanding of the project despite efforts to communicate its goals and modalities.

In discussing the degree to which the project’s objectives were shared between the various groups involved in the project, questions about the project’s scope were raised by respondents from all of the groups that were interviewed. Respondents described how the project’s exclusive focus on primary health care and medications brought attention to other areas of need for the indigents which remained unaddressed. It was also suggested that the project raised some community members’ hopes that help in these others domains might also be forthcoming.

The limits of addressing a single area of need are illustrated by the following scenario that was reported by several respondents: With the project, the indigents could now see the nurse and be prescribed medications for free. However for many medications the nurse instructed the person to take it with each meal, an impossibility for most indigents who did not have consistent access to food. An indigent underlined that medication alone was insufficient to promote wellbeing: “If I’d had to pay when I got sick last year, I would have died. I didn’t have enough to eat, never mind to get medical care.” A member of the VSC also described this reality: “On the other hand, the indigents come and tell me that I helped them get healthy again, but they’re not eating, and so it’s as if they’re still sick.” The project thus targeted an area of important need and drew attention to the indigents within their local communities. It also raised questions about other areas of pressing need, including ones impacting on health. Respondents from across the spectrum of the project raised questions related to these diverse needs, however several of the researchers also expressed that while a broader reach to the project would have been preferable it was not possible due to the restrictions on funding. Moreover, the project was developed to test a process of community selection and demonstrate the feasibility of enacting provisions in the national health policy to provide healthcare user fee removal for the indigents which had not been previously implemented.

The needs of the indigents for adequate food, clothing or shelter went unaddressed, yet the project raised expectations for some that these concerns would also receive attention. Reflections around the project’s focus on healthcare user fees illustrate divergent perspectives from within and outside local communites about the scope of the project, draw attention to the diverse set of needs for the indigents, and suggest one of the limits of the participatory approach.

### Community participation and the risk of stigmatization

The Ouargaye project aimed to test methods to identify the most needy individuals within local villages and provide them with free access to healthcare. Respondents offered various accounts of the nature of vulnerability and how it related to the selection of the indigents. In particular, social isolation and an inability to attend to one’s own needs were emphasized. A woman described why she and her husband were selected as indigent since there was “no one to help us.” Another respondent selected as indigent reported, “we have nothing to eat and no more strength for farming.” The indigents did not have access to healthcare as they could not afford to pay the fees and did not have support from others who could bear such costs. These individuals were thus the poorest individuals in a context, as described by a decision-maker, where “the level of poverty is quite high and therefore the question of resources [to support healthcare access] is sensitive.”

Many respondents discussed the relationship between the selection of the indigents and experiences of marginalization and social relations in the villages in a context “where everyone knows each other.” Members of the research team and some decision-makers reported that one of their primary concerns in the planning phase of the project was the risk that there would be negative consequences of categorizing some as indigent. They reported a particular concern regarding the potential for the selection of the indigents would stigmatize these individuals, and that the participatory model might contribute to this risk. A health worker described that the term ‘indigent’ itself might be negatively viewed: “In the very beginning, there were some people who were reticent because when we say ‘indigent’ , based on the word itself, it’s like a kind of insult.” That other community members selected those who were indigent, and that those selected then received an identity card as an indigent, heightened the concerns of stigmatization.

When interviewed after completion of the project, respondents from across the different groups did not think that the project led to increased stigmatization for those selected. None of the respondents who were selected as indigents reported that they felt stigmatized as a result, though one suggested that some individuals who were not selected felt jealous that she had access to free healthcare and they did not. Discussing the possibility of stigmatization of the indigents, a health worker asserted that though the term indigent might function as a “label” within the village it did not impede the integration of individuals within the community, beyond their existing state of isolation. Indeed, it was suggested that selecting some individuals as indigent may have formalized what was already commonly known and accepted as the established pattern of social relations in the community. This idea relates to the observation of another health worker that following the selection of the indigents:

… everyone congratulated each other and they were proud to see that a certain person had been taken on as indigent. People were not embarrassed and were not treated any differently because of having been selected as indigent or not…

Several respondents described that the project did have impacts on social relations in others ways. A member of the MC reported that in his village the project:

… allowed us to build stronger bonds and become closer. It helped people get to know each other better. The indigents themselves knew that, before this project, the community had compassion for them, but it was just that people didn’t have the means to help them.

A health worker also described that the project “…reinforced the villagers’ interest in the situation of indigents.” One of the indigents also described how the project might influence his actions towards others in his community stating that since he had been helped by the project he would “help others too so that their situation can improve.” These descriptions relate to more general statements by several respondents from the local communities who associated the project with enabling villagers to act in solidarity with their neighbors especially by providing a means to help those who were indigent.

One of the considerations that was raised by serveral respondents was the possibility that the situation of the indigents would change, and that they would no longer need to receive user fee removal. It was generally viewed that in this setting, the situation for most indigents was stable, however it was expressed that there would need to be a means of adjusting the list of the indigents in light of changed circumstances such as if a relative who had moved away would return and be able to take care of the family member.

### Impacts of local funding of user fee removal

The funding mechanism employed in the action-research, subsidizing user fee removal for the indigents through marginal profits of the local pharmacy, located financial responsibility within the local villages and was thought to be more sustainable as it would not rely upon external funding. This model also placed a financial burden, and a degree of risk for the financial integrity of the local pharmacies, upon local communities. The funding model of the project located additional control of the project in the hands of community members. Without the support of the MCs in each village, the project could not proceed.

The use of endogenous funding played a key role in promoting community participation – it also placed significant influence into the hands of the local communities, and contributed to a feeling of “ownership” of the project. This design made the implementation of the project in each village contingent on whether the MCs of the health centers would support the initiative. These groups held multiple and competing responsibilities: they were directly involved in the selection of indigents as they were called upon to validate the final list of selected indigents in their village, yet also were expected to ensure the financial integrity of the local pharmacies from whose budgets the care of the indigents would be subsidized. These responsibilities pulled in opposite directions and had to be negotiated by each MC. There were different responses. For example, one of the MCs refused all individuals from the list presented by the VSCs in the first year of the project, apparently due to concern for the impact on the finances of their pharmacy. However, once this concern was allayed by demonstration that the subsidization of user fees for indigents in other communities did not harm the financial stability of their pharmacies, these same MCs accepted some as indigents in subsequent years.

The funding model, however, also directly contributed to the most frequently expressed reservation about the project by respondents across the groups interviewed: the limited numbers of indigents who were ultimately selected in the participating communities (the equivalent of 1% of the population). A member of an MC reported that the committee’s concern for the viability of the budget for the village dispensary restricted the number of indigents who were eventually selected: “We take this number, because if the number is too high, the [dispensary] won’t be able to function and even those who have money won’t get any products” because the pharmacy will not be able to restock. A member of the research team observed that “When the people in the community realize they are the ones who will carry the cost, then they are much more careful about the selection.” A diverse range of respondents reported that the dual responsibility of the MCs for ratifying the list of indigents and for oversight of the pharmacies led to a restricted selection due to financial concerns despite there being other people in the villages who might legitimately have been considered indigent based on the selected definition of indigence.

The selection and funding models meant that the community held significant control over the project and their active participation was required for its success. However, the use of endogenous funding led to fewer individuals being selected as indigent leaving many vulnerable individuals without the benefit of free healthcare.

### Efforts to promote fairness in the selection of the indigents

In designing the project, researchers and policy-makers were preoccupied with whether the participatory model would reliably identify the worst-off, and whether the process could be co-opted to benefit particular individuals, and to the exclusion of others with genuine needs. To promote an effective and fair selection process a number of steps were implemented including a multi-layered selection process (VSC, local chief, MC) and constrained roles for traditional leaders. Some additional features were included in the design of the project that aimed to promote equity, and that were championed by the research team. Notably, the composition of the VSCs was guided by a concern for gender equity, including a requirement that at least 3 of 7 members be women. The success of this strategy was contested, however, and respondents expressed different views on its impact. Some felt that since the women who were chosen for the VSCs were very active in the social and political life of the community they were able to voice their opinions, however others reported that it was difficult for these women to express dissenting views in front of their husbands or other men in the village. Respondents did not report concern with the gender distribution of those who were identified as indigents.

The effects of the participatory model and the role of traditional leaders in the project were discussed by many respondents. Public support of the project by village chiefs was seen as crucial for community members to be involved, and a key source of legitimacy for the project. This view was identified by respondents from the local communities, as well as by researchers and policy-makers. However, respondents who were researchers and policy makers generally shared the goal of ensuring that village chiefs would have an essentially symbolic role and not be in a position to influence the actual selection of the indigents. A policy maker described this in relation to the need to respect local customs: “… we told ourselves that, for the project to be accepted on the cultural level, the key decision-makers, the opinion leaders, would need to be won over to the cause.” A member of the research team reported: “In a way, we overstepped the social traditions, which we took on, because normally it was the chiefs who should have done it… The idea of including them was to say that we are aware of their role in the community. We can’t leave them out, but we did what we could to minimize their influence in the process.” When asked about the role of the chief, respondents who were from the villages generally viewed the traditional leaders’ participation in ratifying the lists produced by the VSCs as contributing to the quality of the selection, and as reassuring the communities about the value of the project.

An underlying question related to the possibility that certain groups would be benefited but not others, such as if members of a particular ethnic group would be chosen, but not those from other groups. A few instances were reported by respondents when individuals attempted to deflect the selection process away from identification of those who were truly most needy. For example, a member of the research team reported: “… there’s one village where it didn’t work at all, because the chief wanted to include even his children as indigents and the [MC] refused. That was a flagrant case. They didn’t understand their role.” This situation appears exceptional. Reflecting on the composition of those identified as indigent across the district, a member of the district leadership team reported that the selection did not reflect discrimination between groups: “… when we look at some of the indigents selected, for example, we see there are all kinds of people: Christians, Muslims, apoliticals, and even some people who used to be important but aren’t any more.” Despite being a source of concern in planning the project, respondents did not think that efforts to distort the selection process had succeeded, nor were they perceived as common, having mostly occurred at the beginning of the project and were adequately addressed by the multiple layers of ratification which resulted in increased accountability.

### Power relations and the development of effective partnerships

The Ouargaye project was predicated upon the coordinated action of stakeholders crossing multiple spheres. As well as community participation, the project required collaborations among members of the research team and local decision-makers and practitioners, among national level policy-makers and researchers, and between researchers from Canada and Burkina Faso. These relationships, and the degree of collaboration and partnership created and sustained within them, shaped how the project was implemented and evaluated, as well as potential for scale-up and sustainability of the intervention. They were also associated with ethical concerns, especially when objectives and expectations did not align or there was significant inequality of power between partners.

A policy-maker described effective partnerships being based on mobilizing the different expertise or contributions of partners toward a shared purpose. He noted that a successful partnership “… makes up for what the other party lacks, it’s a give-and-take encounter.” Several respondents described situations where partners brought different but mutually reinforcing skills, interests and knowledge in ways “[t]hat were useful for both sides.” A sense of commitment and responsibility for the project was described as crucial for the engagement of all stakeholders. A policy-maker thus emphasized that local actors needed to have a stake in decision-making: “It’s quite important that [community representatives] feel involved at the decision-making level, that they participate in decisions before they are getting involved in the implementation.” Several members of the research team and policy-makers reflected that achieving effective partnerships was crucial for the project’s success, and suggested that partial or limited partnerships accounted for some of the difficulties encountered.

Unequal power and influence were present at many levels of partnership. Questions related to power were most commonly raised by respondents in relation to policy-makers, and within the research team. For example, research team members from Burkina Faso discussed constraints associated with partnerships between local and international researchers. A member of the research team reported the following dynamic:

The challenges are that, in every case, it’s Northern researchers who are the program leaders, it’s their project, they’re the ones who wrote the protocol, and now, when it comes to the implementation, the intervention on the ground, we’re the ones who are there, who make it happen…

This distribution of responsibilities was identified as resulting from different expertise of the partners but also from the fact that funding from Canada was supporting the project: “It’s bound to create dominance, because when we talk about a ‘leader’ , that’s the person with the money, that creates dominance…” Respondents described these financial arrangements as an impediment to a more balanced partnership, and an issue that needed to be navigated by all members of the research team.

Another site of challenge in establishing effective partnerships was between researchers and national policy-makers. Several respondents directly associated the quality of these partnerships with the broader success of the project. A preoccupation for many respondents, including some research team members, policy makers and health workers, was the potential for the user fee exemption to be sustained in Ouargaye, to be scaled-up in other regions of Burkina Faso, and to influence policy setting in other countries. A member of the research team described that a key goal of the project, “…was to make sure that it wouldn’t be just an exercise in producing evidence, but that it would find concrete expression in the national health policy in terms of its application.” Effecting policy change was seen as uncertain, however, in part due to the variety of institutional actors involved, and barriers for partnering between them, including the role of multiple government ministries. Since the project’s focus, the needs of the indigents, fell at the overlap of the health and social services sectors respondents described that ensuring the engagement of officials from these ministries was more difficult as a result.

Partnership development was also limited where priorities were not shared. A research team member questioned the degree to which the project’s goals matched the preoccupations of national level decision-makers:

… most of the time decision-makers at the central level are in positions that are much more political and they don’t share the researchers’ concern for the research results. So, the conclusions and results from the field have little influence on decisions taken at the central level.

Several policy-makers, however, described that the impact on policy, including scale-up, would have been enhanced with more sustained communication of how the project was unfolding. Due to these factors, many respondents, including both researchers and policy-makers, viewed these partnerships as only partially realized, and thus limiting the prospects of sustainability and scale-up.

## Discussion

This action-research project was carried out in a largely traditional, rural and impoverished district of Burkina Faso. While the policy implemented in the communities – removal of healthcare user fees for those selected as indigent – directly benefited a small percentage of the population, the research process engaged many within and beyond the community, and had impacts on social relations within the villages. The logic and principal goals of the project aimed at promoting equity in access to healthcare services for a disadvantaged group, itself an ethical concern, based on a judgement that inaccessibility of healthcare for the poorest members of a society is unjust [34]. The goal of increased access for the indigents, shared by researchers and policy-makers involved in the project, was also consistent with the national health care policy of Burkina Faso [11]. For these actors, the project in Ouargaye district was conceived as an opportunity to demonstrate feasibility of identifying the indigents and funding free healthcare services, and thus developing evidence to support equity-enhancing policy change that would be sustained locally and scaled up to other regions of the country. These broad goals, however, become concretized in the implementation of the project as small-scale case studies in the villages of Ouargaye district, where conceptions of equity and vulnerability are differently understood, and implemented in a context where social gradients may be viewed by local communities as an inherent part of social life and the proper order of the community [35].

Implementation of the action-research took place within the local social worlds of these communities. As illustrated by the descriptions of the respondents, the project was also associated with risks and burdens for communities and individuals, as well as a range of potential benefits. In all participatory research models, implications for communities are of primary concern and require attention alongside protections for individual research participants [36]. While the equity-oriented goals of helping the worst-off may seem of great urgency in the Ouargaye action-research, a range of important ethical issues are raised. Drawing upon analyses of ethical issues in community-based participatory research [28,36] and action research [18,37], we discuss the relevance of the following issues to this project: balancing risks and benefits for communities and individuals, respect for communities and efforts to promote fairness, social justice concerns, dynamics of power and privilege, navigating the politics of policy-making, and development of relationships of mutual respect.

### Balancing risks and benefits for communities and individuals

In planning the participatory model of the project, much discussion took place between researchers and policy-makers regarding how to respect local communities and local values, for example through engagement of local leadership, while ensuring equity in the selection of the indigents. A primary consideration of traditional models of research ethics relates to the requirement to demonstrate respect for respondents in research. Respect for communities has emerged as a related principle for researchers to uphold and is especially pertinent in community-based and participatory research [28,38]. In close-knit, communally-oriented communities such as the villages of Ouargaye district, considerations of respect for individuals and communities are closely intertwined. Thus, as Brydon-Miller describes in relation to action-research, “researchers must remain mindful of the complex nature of balancing individual and collective action and the relationships of power and privilege which inevitably frame these processes of decision-making.” [37]. In this project, a risk that those selected as indigent would be stigmatized was identified and, while seeking to minimize the risk, evaluated in relation to the benefits of increased access to healthcare services for these individuals. Likewise, the impact of the participatory model, including associated burdens for community members and the possibility of social discord, were considered in light of the potential benefits. The reports of respondents suggest that the more significant potential harms of this project, including stigmatization, social discord and financial problems for local pharmacies, were minimized or avoided by the strategies undertaken in the project [39].

### Respect for communities and efforts to promote equity

Decisions related to how to act upon the principle of respect for communities raise challenges, perhaps particularly so in the context of global health research, including how to balance respect for community values with other commitments. Two primary issues encountered in this project relate to the roles accorded to the traditional chiefs and questions of gender equity. The involvement of traditional leaders in research decision-making may be crucial for the success of research and affords legitimacy to the research process [40]. It also demonstrates respect for community values. However, in this setting researchers and policy-makers sought to limit the influence of the chiefs in order to minimize the possibility that selection of the indigents benefited certain groups or families over others. Thus a trade-off between respect for community values and equity was undertaken. In the second example, the Canadian researchers – in part guided by requirements of the research funder – insisted that 3 out of 7 members of the VSCs were women. While this was experienced as an outside imposition, it was implemented in each VSC and seemed to have been generally accepted by those interviewed for the evaluation. This contrasts with other settings where external requirements of equal distribution of men and women on committees were not heeded [41]. In reality, those selected as indigent were evenly split between women and men though it is impossible to know if a similar distribution would have resulted if committees had been entirely composed of men. The ethical analysis of research commonly requires the weighing of competing moral claims and commitments [42]. The principle of respect for communities is no exception, and the Ouargaye project design reflects this process of evaluating competing values.

### Social justice concerns

A distinctive aspect of this project was the reliance on funding from within local communities to pay for costs associated with the user fee removal. This feature of the project was consistent with the national health policy which directed that user fee removal for the indigents be subsidized by such a mechanism. It was also suggested that this approach would be less precarious and more likely to be sustained over time than support from nongovernmental agencies. From this perspective, this funding approach thus supports a greater chance of longer term support for the indigents in these communities. The discourse of respondents suggests that the endogenous funding was also associated with feelings of solidarity between community members, and reinforced the communities’ feelings of ownership over the project. Endogenous funding can be questioned on several levels however. First, this model places financial burdens onto impoverished communities when, it can be argued, that ensuring access to care for those vulnerable should be a governmental responsibility. Second, this funding scheme had the effect of limiting the numbers identified as indigents and thus able to benefit from user fee removal. Given the widespread level of need in these communities, including more individuals as indigents would have gone further to addressing barriers in accessing health care services. It seems reasonable to suggest that social justice considerations can be used to argue for and against the funding model utilized in the project.

### Power and privilege

Issues of power and influence are unavoidable in participatory research [43]. While the Ouargaye project was initially conceived by researchers and policy-makers, local communities were key decision-makers in giving shape to the particular mechanisms of the action components of the project and assumed a range of responsibilities for its implementation in the villages. As well as the risks described earlier, they also bore burdens including sitting on committees, organizing evaluation visits, as well as other practical tasks, typically without remuneration. The involvement of these individuals was necessary for the project though it can be asked how free these individuals felt to decline requests for assistance as the project unfolded. Questions of voluntariness, in this case, not only of the indigents but also other local partners, such as health workers and VSC members, are common in action-research [44]. Due to the extensive involvement of the community– including funding the user fee removal through the dispensary profits – there were greater opportunities for the community to shape how this project would develop in light of their values and social structures, and was associated with feelings of ownership over the project. This involvement, however, was principally focused on the action component of the project in comparison to the evaluations. It is worth noting that those eventually identified as indigents through the community selection were unlikely to have been those in the community who contributed to its design and implementation. Their social isolation and destitution which resulted in their identification as indigents limited opportunities to contribute to project planning despite efforts by researchers and community leaders to solicit the involvement of diverse members of the community. This situation raises concerns related to the absence from these processes of the perspective of those individuals who are most directly impacted by the project. It also suggests how local social hierarchies and privilege influenced the extent and reach of the participatory approach.

### Navigating politics of policy-making

Though geographically situated in the villages of Ouargaye district, the scope of the project extended far beyond these communities. The project involved a loose series of concentric circles of partnership and activity. At a broad level, researchers from Burkina Faso and Canada were involved in the initial conception of the project. Policy-makers at the national level were then involved in evaluating the value of the proposal for national priorities and facilitating the project implementation in a local setting. At the local level another domain of participation was opened up: collaborative efforts between researchers, health workers and communities to establish and implement mechanisms to identify the indigents and subsidize their healthcare fees. The ambition of the project was that the results would travel back through these concentric circles – from village, to region, to nation, in order to influence policy setting and allow sustainability of the model and scale-up nationally. This description of layers, and movement of ideas, information and resources between them, emphasizes the relational and political complexity of the project. In one sense, it highlights the importance of the political nature of action-research that aims to influence the policy landscape beyond the research site. As Williamson and Prosser have argued, action-research is inherently political [45]. Researchers must be “politically astute” in order to navigate these processes [46] and to promote the likelihood that research outcomes will contribute to social value through integration in policy setting measures [42].

Though yet to be scaled up at a national level, the results of the action-research in Ouargaye have led to efforts to expand user fee removal for the indigents in Burkina Faso. In 2013, the World Bank announced plans to implement a project based on the community selection model developed in the Ouargaye project that will be carried out in 12 districts of Burkina Faso, and to finance user fee removal for 20% of the population. Given its reliance on external funding, the sustainability of this intervention is uncertain. However, it represents an important initiative to address barriers to health care access.

### Relationships of mutual respect

Beyond the micropolitics of action-research, these realties also highlight the inherently relational nature of this project. Community-based and participatory models of research are built upon relationships [18]. It follows that ethical issues in these relationships will be of vital importance. Partnerships in global health research have received increased attention [47]. It can be very difficult to develop equitable and respectful partnerships. Risks include the creation of superficial partnerships that fail to demonstrate mutuality and common purpose, and which may be created for instrumental purposes such as securing funding or ethics approval. Multiple layers of relationships were created in the Ouargaye project. The degree of mutuality encompassed by these relationships varied and was influenced by different factors. For example, mutuality in the relations between researchers from Burkina Faso and Canada was shaped by the role of funding models and its impact on leadership and decision-making. Likewise, different professional roles and responsibilities between policy-makers and researchers were factors that led to limitations in the partnerships that were created.

Reflecting upon the Ouargaye project and the challenges of promoting respectful relationships across the many sets of relations that were developed in the project, we would suggest two approaches that would strengthen mutuality. The first is careful attention to the influence of privilege and position. Relationships in global health and participatory research will be strengthened by the development of what Iris Marion Young terms “asymmetrical reciprocity.” Young argues that moral respect requires deliberate recognition of asymmetries due to differences in social position and power, and the development of trust and reciprocity [48]. This reciprocity of respect, trust and consideration does not gloss over differences but entails careful and deliberate attention to the situatedness of partners, including explicit attention to differences in culture and life experience. Second, acknowledgement that ethical considerations will be understood differently by others, and a willingness to engage respectfully with these divergent perspectives, is crucial for relationships of mutual respect. These two approaches are important aspects of respectful research relationships, and are particularly important in participatory research in global health. The relational ethics of participatory and action-research will benefit from further exploration, including attention to how relational conceptions of personhood and solidarity can help guide research planning and implementation [18,49].

### Limitations of the study

A number of limitations are relevant to the findings reported in this article. First, the structure of the data collection was an intensive 3-week period of interviews. The fact that all interviews were conducted prior to transcription or initiation of analysis did not allow for a recursive approach between data collection and analysis, nor theoretical sampling. Further insight would also have been gained from the opportunity to conduct interviews at multiple stages of the action-research. This concern was allayed somewhat due to the participation of one of the authors (VR) who was involved in the project from its inception and was able to situate the interviews within the broader history of the project. Additionally, interviews with indigent individuals were conducted in the local language, Yana, by a local research assistant and translated to French. A further challenge relates to the distinction between action and research phases in the analysis. The difficulty of cleanly distinguishing these components of the project is directly associated with the logic of the action-research approach which involves breaking down these categories [24].

## Conclusion

This examination of the Ouargaye project serves to illuminate the distinctive ethical terrain of a participatory public health action-research project, including considerations of vulnerability, respect for communities, collective risk, equity, power, participation, partnership and social justice. As Shore and colleagues describe in relation to Community-Based Participatory Research, the ethical implications of such research exceed research ethics’ “traditional framework of ethical analysis to include community-level and partnership-oriented considerations” [36]. Participatory and action-research projects require careful attention and effort to establish and maintain relationships that are characterized by mutual respect, and attention to power and privilege. Several approaches may help to support this objective, including humility and engagement on the part of researchers and other actors, and the development of asymmetrical reciprocity between partners. As future projects to remove healthcare user fees for those identified as indigents are developed, it should also be considered how other determinants of health might be addressed to further promote the wellbeing of these individuals.

## Competing interests

The authors declare that they have no competing interests.

## Authors’ contributions

MH and VR developed the original study design. MH and PG were responsible for data collection. MH and PG conducted the data analysis, which was subsequently reviewed with VR. MH wrote the manuscript with contributions from VR and PG. All authors approved the final manuscript.

## Pre-publication history

The pre-publication history for this paper can be accessed here:

http://www.biomedcentral.com/1472-6939/15/13/prepub
